# Pervaporation Polyurethane Membranes Based on Hyperbranched Organoboron Polyols

**DOI:** 10.3390/membranes12121247

**Published:** 2022-12-09

**Authors:** Ilsiya M. Davletbaeva, Oleg O. Sazonov, Sergey E. Dulmaev, Alexander V. Klinov, Azat R. Fazlyev, Ruslan S. Davletbaev, Sergey V. Efimov, Vladimir V. Klochkov

**Affiliations:** 1 Technology of Synthetic Rubber Department, Kazan National Research Technological University, 68 Karl Marx str., Kazan 420015, Russia; 2 Department of Chemical Process Engineering, Kazan National Research Technological University, 68 Karl Marx str., Kazan 420015, Russia; 3 Material Science and Technology of Materials Department, Kazan State Power Engineering University, 51 Krasnoselskaya str., Kazan 420066, Russia; 4 Institute of Physics, Kazan Federal University, 18 Kremlevskaya str., Kazan 420008, Russia

**Keywords:** pervaporation membranes, polyurethanes, water vapor permeability, isopropanol dehydration

## Abstract

On the basis of aminoethers of boric acid (AEBA), polyurethane vapor-permeable and pervaporative membranes were obtained. AEBAs, the structure of which is modified by bulk adducts (EM) of diphenylol propane diglycidyl ether and ethanolamine, were studied. It turned out that AEBA exists in the form of clusters, and the use of EM as a result of partial destruction of associative interactions leads to a significant decrease in the size of AEBA-EM particles and their viscosity compared to unmodified AEBA. The introduction of EM into the composition of AEBA leads to a threefold increase in the vapor permeability of polyurethanes obtained on their basis. The observed effect is explained by the fact that a decrease in the size of clusters leads to loosening of their dense packing. Areas of clustering due to associative interactions of hydroxyl groups, together with the hydrophilic nature of polyoxyethylene glycol, create channels through which water molecules can penetrate. The increase in vapor permeability is accompanied by a multiple increase in the permeability coefficients in the pervaporative dehydration of isopropanol.

## 1. Introduction

Pervaporation is a unique process among membrane separations. The process allows the separation of miscible liquids of similar molecular weight and is an alternative to distillation. However, the relative cost of membrane devices compared to distillation equipment means that in most separation applications where distillation works efficiently, pervaporation is not a viable alternative. In addition, pervaporation is generally not economically viable as a multi-stage separation process. Pervaporation can be a useful technique when separating or removing a small amount of a single liquid from a liquid mixture [1,2,3,4,5,6,7,8,9,10].

The use of the pervaporation process is most appropriate when separating liquid mixtures that form azeotropes, where vapor and liquid have the same composition in equilibrium and standard distillation cannot provide their separation. There are many mixtures of organic solvents with water that have azeotropic compositions. Pervaporation is generally economical at water levels of about 10% or less, with achievable water levels in the final product ranging from hundreds to 10 ppm. The center of the entire separation process is the membrane [11,12].

Pervaporation membranes are currently available on the market and are made from a wide variety of materials that include polymeric, inorganic, and hybrid materials. However, polymers remain the most versatile and efficient candidate among them due to ease of fabrication as well as relatively low production costs compared to inorganic and hybrid materials [13]. Hydrophilic polymers such as polyvinyl alcohol, polyacrylic acid, chitosan, cellulose, and alginates were used to dehydrate solvents in early generation membranes due to their predominant affinity for water [14,15,16].

However, these membranes are subject to swelling, which leads to a decrease in separation efficiency and a decrease in mechanical strength. Therefore, these membranes require additional modifications, such as crosslinking, chain grafting, copolymerization, and addition of adsorbing materials to improve their stability and selectivity [17,18,19,20]. Meanwhile, rigid chain glassy polymers such as polyimide, polyamide, polybenzoxazole, polybenzoxazinone, and polybenzimidazole have become potentially applicable membrane materials for this industry because their rigid structure positively affects not only diffusion selectivity, but also improves swelling resistance and durability [20]. However, the use of these polymers in solvent dehydration is compromised by their hydrophobicity and low selectivity.

Pervaporation membranes can be either organoselective (organic substances preferentially permeate through membranes) or water-selective (water preferentially permeates through membranes), which is set by choosing the material and modifying it during the membrane fabrication [21,22]. Promising for the manufacture of membrane materials are polyurethanes, since they are an example of the most widely used universal polymers [23,24,25,26,27,28,29].

Polyurethane membranes based on hydroxyl-terminated polybutadiene, 4,4-dicyclohexylmethane diisocyanate, and 1,4-butanediol were synthesized in [30]. The best pervaporation characteristics during the dehydration of isopropanol (10 wt.% water) were achieved at NCO/OH = 1.5 at a temperature of 30 °C (320 g/m^2^·h, selectivity was 180). In [31], polyurethanes were modified by immersing polyurethane membranes in a mixture of formic acid and hydrogen peroxide in a 1:1 molar ratio. The best pervaporation characteristics during the dehydration of isopropanol (10 wt.% water) were achieved at a temperature of 25 °C (1583 g/m^2^·h, selectivity was 4.7). In [32], it was proposed to increase the resistance of a polyurethane membrane to solvents and the characteristics of pervaporation due to spatial crosslinking with methyl methacrylate. The best productivity was 365 g/m^2^·h and the selectivity was 212 in the pervaporative separation of isopropanol (10 wt.% water) at an operating temperature of 30 °C. In [33], a polyurethane membrane was developed based on 2,4-toluene diisocyanate and polyethylene glycol (PEG) with different molecular weights. PEG molecular weight has been found to have a significant effect on the selectivity and performance of polyurethane membranes. The polyurethane membrane based on PEG-4000 showed the best performance in isopropanol/water (20 wt.% water) separation at 30 °C, demonstrating a productivity of 730 g/m^2^·h and a selectivity of 13.

However, many polyurethanes based on low molecular weight polyols have low tensile strength at room temperature. An effective method to solve this problem is cross-linking. Hyperbranched polyols in this way can be used as crosslinkers [34,35]. Such branched structures are characterized by remarkably low intrinsic viscosities and high solubility, resulting in good compatibility with parent systems. Hyperbranched polyols attract attention due to the abundance of synthesized structures, unique molecular architecture, and wide range of properties [36]. They have a spherical shape and a high density of functional groups [37]. Considering their unique thermal, mechanical and rheological properties [38], in combination with good dissolving power, hyperbranched polymers based on such polyols are used in coatings [39,40,41] drug transport [42] and pervaporation [43,44,45,46,47]. Compared to dendrimers, hyperbranched polyols are relatively easy to synthesize and are suitable for large scale production.

In connection with the above, polyurethanes obtained on the basis of hyperbranched organoboron polyols represent a prospect for further research in this area [48,49,50]. Studies in the field of the synthesis of organoboron polymers have shown [51] that the products of the interaction of 1,2-, 1,3-diols and aromatic ortho-diols with boric acid and boron chloride are acid ethers that condense at elevated temperature to dimers in a five-membered ring. With an excess of diol, diboratesare formed containing the fragment B (III) -O-R. 1,3-diols in the reaction with boric acid form the most stable unstrained rings linked to each other by a methylene diborate fragment. Heating boron triacetate with glycerol in vacuo gives polymeric triolborates, which are glassy polymers. Tetraols were etherified with boric acid to form polyethers of boric acids [52]. More complex structures can be obtained with two types of acids: orthophosphoric and boric acids [53]. Organoboron oligomers and polymers are used in catalysis [54], to obtain heat-resistant [55], porous [56], luminescent [57] and medical materials [58], polymer electrolytes [59], and as a basis for the synthesis of polyurethanes [60,61,62,63]. An analysis of the literature data shows that the use of borates in polyurethane compositions leads to a significant increase in their compressive strength. The use of boric acid derivatives contributes to the formation of a more ordered structure of polymers and, thereby, reduces the brittleness of materials [62,63,64]. At the same time, the presence of borates does not change the size and shape of pores in porous materials and improves their functional properties [65,66,67].

In our previously published study, hyperbranched organoboron polyols were synthesized as amino ethers of boric acid (AEBA) [48,49,50]. To increase the free volume of the polyurethane matrix, adducts of diphenylolpropane diglycidyl ether and ethanolamine (EM) were introduced into the AEBA architecture [48]. The membranes obtained using such polyurethanes showed high efficiency in the separation of gas mixtures containing ammonia due to the high values of its permeability. It has also been shown that the obtained polyurethanes have relatively high vapor permeability. However, the effect of increasing the permeability of water and ammonia molecules was observed at such a low EM content that it is not able to have a noticeable effect on the architecture of macromolecules. In [48,49], it was also shown that the peculiarity of the supramolecular structure of polyurethanes obtained on the basis of AEBA is pore formation. However, in the same work it was noted that the pores do not affect diffusion processes. In this regard, the mechanism of diffusion of polar molecules through such polyurethanes turned out to be unexplained. In addition, it was shown that the terminal hydroxyl groups of AEBA obtained on the basis of low-molecular-weight glycols (diethylene glycol, triethylene glycol) lose their ability to react with isocyanates due to their involvement in intense associative interactions. In connection with the results obtained and the need to predict further research, it became necessary to explain the causes of the observed phenomena. For this purpose, in this work, we studied the chemical structure of AEBA and the products of their interaction with the EM adduct (AEBA-EM) using ^11^B NMR spectroscopy, the regularities of changes in rheological properties and particle sizes depending on the content of EM in the composition of AEBA-EM. Measurements of the physical and mechanical properties and thermal stability of polyurethanes obtained with different EM content in the composition of the corresponding AEBA-EM-PU were carried out. Infrared spectroscopy was used to determine the effect of associative interactions of AEBA terminal hydroxyl groups on their reactivity with isocyanates. A correlation has been established between vapor permeability and pervaporative characteristics of membranes based on AEBA-EM-PU during pervaporative dehydration of isopropanol.

## 2. Materials and Methods

### 2.1. Materials

Triethanolamine (TEA) was produced by OJSC Kazanorgsintez (Kazan, Russia). Poly (ethylene glycol) (PEG) produced by Nizhnekamskneftekhim PJSC (Nizhnekamsk, Russia). Epoxy resin DER-331 is from Dow Chemical Company. Ethanolamine and boric acid (99.99%) were purchased from Sigma-Aldrich (Merck KGaA, Darmstadt, Germany). Polyisocyanate (PIC) manufactured by Kumho Mitsui Chemicals, Inc. Glycols were dried from traces of moisture at 5 mm Hg and 353 K. CuCl_2_∙6H_2_O was dehydrated at 393 K for 48 h.

### 2.2. Procedure for the Synthesis of Ethers and Aminoethers of Boric Acid

To obtain aminoethers of boric acid, first, 6 mol of boric acid, 3 mol of PEG and the adduct EM in the range of 0.05–2.0 wt.% were mixed in a three-necked round-bottom flask, the reaction was carried out at T = 80 °C for 2 h and a pressure of 10 mm Hg. As a catalyst 0.1 wt% copper chloride (CuCl_2_) was used. Then, 1 mol of TEA and 8 mol of PEG was introduced into the reaction system, and further interaction was carried out at T = 80 °C for 2 h and a pressure of 10 mm Hg. The molar ratio of [TEA]:[H_3_BO_3_]:[PEG] ended up being 1:6:11.

To obtain the ether of boric acid, 1 mol of boric acid and 3 mol of PEG were mixed in a three-necked round-bottom flask, and the reaction was carried out at T = 80 °C for 2 h and a pressure of 10 mm Hg.

### 2.3. Procedure for the Adducts Synthesis (EM)

To prepare adducts, DER-331 and MEA at a molar ratio of 2:3 were used. Ethyl acetate as a solvent was used. The synthesis was carried out in two stages. At the first stage, the calculated amount of DER-331 (2 mol), MEA (1 mol) and ethyl acetate was placed in a two-necked flask, heated under reflux to T = 78 °C and kept for an hour. At the second stage, the remaining amount of MEA (1 mol) was added and heated to T = 78 °C and kept for an hour. At the end of the synthesis, it was poured into a container and the solvent was removed.

### 2.4. General Procedure for the Synthesis of PUs Based on Aminoethers of Boric Acid

To obtain polyurethane film materials, AEBA was mixed with PIC at their mass ratio of 1:1. Urethane formation was carried out on a glass surface at room temperature for 24 h. The thickness of the polyurethane films was 90 microns.

### 2.5. Manufacturing of Composite Pervaporation Membranes

A hydrophilic porous substrate based on a fluoroplastic ultrafiltration membrane (UFFK, pore size 50 nm, thickness 90 µm, produced by Vladipor, Vladimir, Russia) was used for the selective AEBA-PU/AEBA-EM-PU layer. The selective layer was applied to the substrate using a cylindrical applicator KAU-1 (Konstanta, St. Petersburg, Russia). The AEBA-PU/AEBA-EM-PU selective layer was cured at room conditions for 24 h.

### 2.6. Measurements

#### 2.6.1. Dynamic Viscosity and Density Measurements

Using a Stabinger viscometer SVM 3000 (Anton Paar, Graz, Austria), the dynamic viscosity of the samples was measured with an error of 0.00005 mPa∙s.

#### 2.6.2. NMR Spectroscopy

^11^B NMR spectra of samples were recorded on an Avance II 500 (Bruker, Billerica, MA, USA) spectrometer. Operating frequency was 160.46 MHz. Sample temperature was set to 30 °C. A total of 512 scans were accumulated in each measurement with the pulse repetition period of 3.7 s.

#### 2.6.3. Water Concentration Measurement

A Mettler Toledo V20 volumetric titrator (Mettler Toledo, Zurich, Switzerland) based on the Karl Fischer method was used to measure the water concentration.

#### 2.6.4. Fourier-Transform Infrared Spectroscopy Analysis (FTIR)

An InfraLUM FT 08 Fourier spectrometer (Lumex, St. Petersburg, Russia) with a spectral resolution of 4 cm^−1^ and a number of scans of 32 in the attenuated total reflection technique was used to record FTIR spectra.

#### 2.6.5. Tensile Stress-Strain Measurements

Tensile stress–strain tests were carried out using the universal testing machine Inspekt mini (Hegewald&PeschkeMeß und Prüftechnik GmbH, Nossen, Germany) at 293 ± 2 K, 1 kN. Film samples with dimensions of 45 × 15 mm were used. The crosshead speed was 50 mm/min.

#### 2.6.6. Dynamic Light Scattering (DLS)

Particle sizes were measured in disposable plastic cuvettes with an optical path length of 1 cm using the dynamic light scattering (DLS) method. The ZetasizerNano ZS device (Malvern, Worcestershire, UK) is equipped with a 4 mV He–Ne laser and operates at a wavelength of 632.8 nm. The detection angle was 173°, the medium temperature was 25 °C.

#### 2.6.7. Water Vapor Permeability (WVP) Measurements

The ASTM method of E 96-80B was used to measure WVP. For this, cylindrical glass cups 50 mm in diameter and a height of 70 mm were used. The cups were pre-filled with deionized water. Membranes were placed on top of the cups and ensured their perfect seal. A space with a height of 15 mm was left between the membrane and the water surface. The cups were placed in a chamber and kept at temperature of 22 °C and 40 °C for 24 h. Vapor permeability values were calculated using the following formula:

(1) $$WVP=\frac{G}{tA}$$

where *G* is the weight change in grams; *t* is the duration of the test in hours; *A* is the test area in m^2^.

The thickness of the samples was 90 microns. WVP was expressed in units of g/m^2^ per 24 h.

#### 2.6.8. Contact Angle Measurements

Water contact angle was defined as the angle between the tangent drawn to the surface of the wetting liquid and the wetted surface of the solid. The contact angle of wetting is always measured from the tangent towards the liquid phase. Distilled water was used as the liquid. The membrane was placed on a stand in the measuring cell, which was mounted on a table holder. A drop of water in an amount of 1 mm^3^ was applied to the membrane surface. The droplet height h and droplet diameter d were determined using an ocular micrometer.

The value of cos *θ* was calculated according to:

(2) cos θ=d22−h2d22+h2

#### 2.6.9. Water Absorption Measurements

The method of water absorption is based on the gravimetric determination of the amount of water which is entrapped in an elemental sample.

#### 2.6.10. Pervaporation Processes

**Scheme 1:**
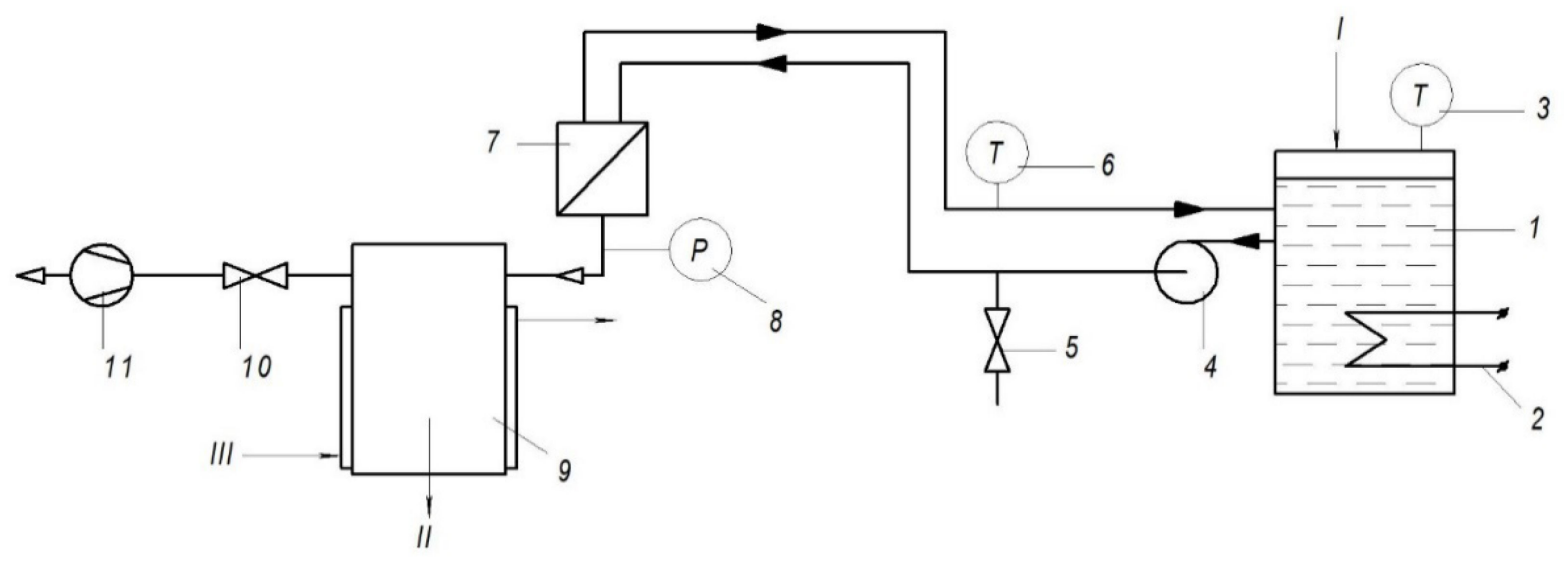
Schematic representation of the setup for the pervaporation equipment: 1—container for raw materials; 2—electric heater; 3, 6—thermometers; 4—recirculation pump; 5, 10—valve; 7—membrane cell; 8—vacuum pump; 9—cold trap; 11—vacuum gauge. I—feed stream, II—permeate, III—refrigerant.

Experimental studies to determine the permeate flow and separation factor of polyurethane membranes were carried out on a laboratory pervaporation unit shown in Scheme 1. The pervaporation process was carried out for a mixture of isopropanol/water at a concentration of 85/95 wt.% isopropanol and 15/5 wt.% of water in the model mixture, at a temperature of 40 °C/60 °C and a vacuum in the permeate part of 20 mm Hg. The effective membrane surface was 75 cm^2^. The rate of movement of the retant and the pressure in the permeate region were the same in all experiments. In all cases, it was assumed that mass transfer through the membrane is carried out by a diffusion mechanism. In this regard, the solution-diffusion model was used.

A model mixture of isopropanol/water was prepared from demineralized water (specific conductivity 5 μS/cm) obtained on an Osmodemi 12 unit (ERREDUE S.p.A, Livorno, Italy) and absolute isopropanol with a main component content of 99.95 wt.% (Sintez Spirt LLC, Orenburg region, Orsk, Russia). The concentration of the model mixture was controlled using a volumetric V20 and coulometric C20 titrators (Mettler Toledo co. Ltd., Greifensee, Switzerland).

Conducting experimental studies began with the fixation of the studied pervaporation membrane in the membrane module 7. Then, the model mixture in a volume of 5 L was poured into container 1. Turning on the recirculation pump 4 and the electric heater 2 reached the required temperature for the process. The temperature of the model mixture was measured and controlled using thermometers 3 and 6, respectively.

After reaching the set temperature, the vacuum pump 11 was turned on; this was the beginning of the pervaporation process. The vacuum depth was controlled and measured using a valve 10 and a vacuum gauge 8. Vapors that passed through the membrane were trapped in a cold trap 9 cooled to −80 °C by a refrigerated bath circulator CRYO-VT-05-02 (TERMEKS LLC, Tomsk, Russia). The process of pervaporation separation was carried out from 3 to 5 h. Then, the amount of permeate that was in the trap was weighed on an AJ-1200CE balance (SHINKO DENSHI Co., Ltd., Tokyo, Japan), and its composition was measured on a Crystal 5000 gas chromatograph (JSC SDO «Chromatec», Yoshkar-Ola, Russia). The gas chromatograph was equipped with a thermal conductivity detector and an HP-FFAP 50 m × 0.53 mm × 0.25 µm capillary column (Agilent Technologies, Inc., Santa Clara, CA, USA). The concentration of the initial mixture during the entire experiment was considered unchanged due to the small amount of permeate formed. The validity of this assumption was controlled by analyzing the amount of water in the mixture taken from valve 5 using titration.

The values of the total permeate flux $\bar{J},$ the separation factor *α*, and the pervaporation separation index PSI were determined from the measurement data using the following expressions:

(3) $$\bar{J}=\frac{m^{p}}{F\Delta t}$$

(4) $$\alpha =\frac{{\bar{x}}_{A}^{P}/{\bar{x}}_{A}^{F}}{{\bar{x}}_{B}^{P}/{\bar{x}}_{B}^{F}}$$

where $m^{p}$ is the mass of the permeate, kg, collected over the time interval Δt, h.; F is the membrane surface area m^2^; ${\bar{x}}_{A}^{P}$ and ${\bar{x}}_{B}^{P}$ are the mass concentrations of the components A (isopropanol) and B (water) in the permeate, respectively, wt.%; ${\bar{x}}_{A}^{F}$ and ${\bar{x}}_{B}^{F}$ are mass concentrations of components A and B in retant (raw material), respectively, wt.%.

## 3. Results

### 3.1. Boric Acid Etherification

**Figure 1:**
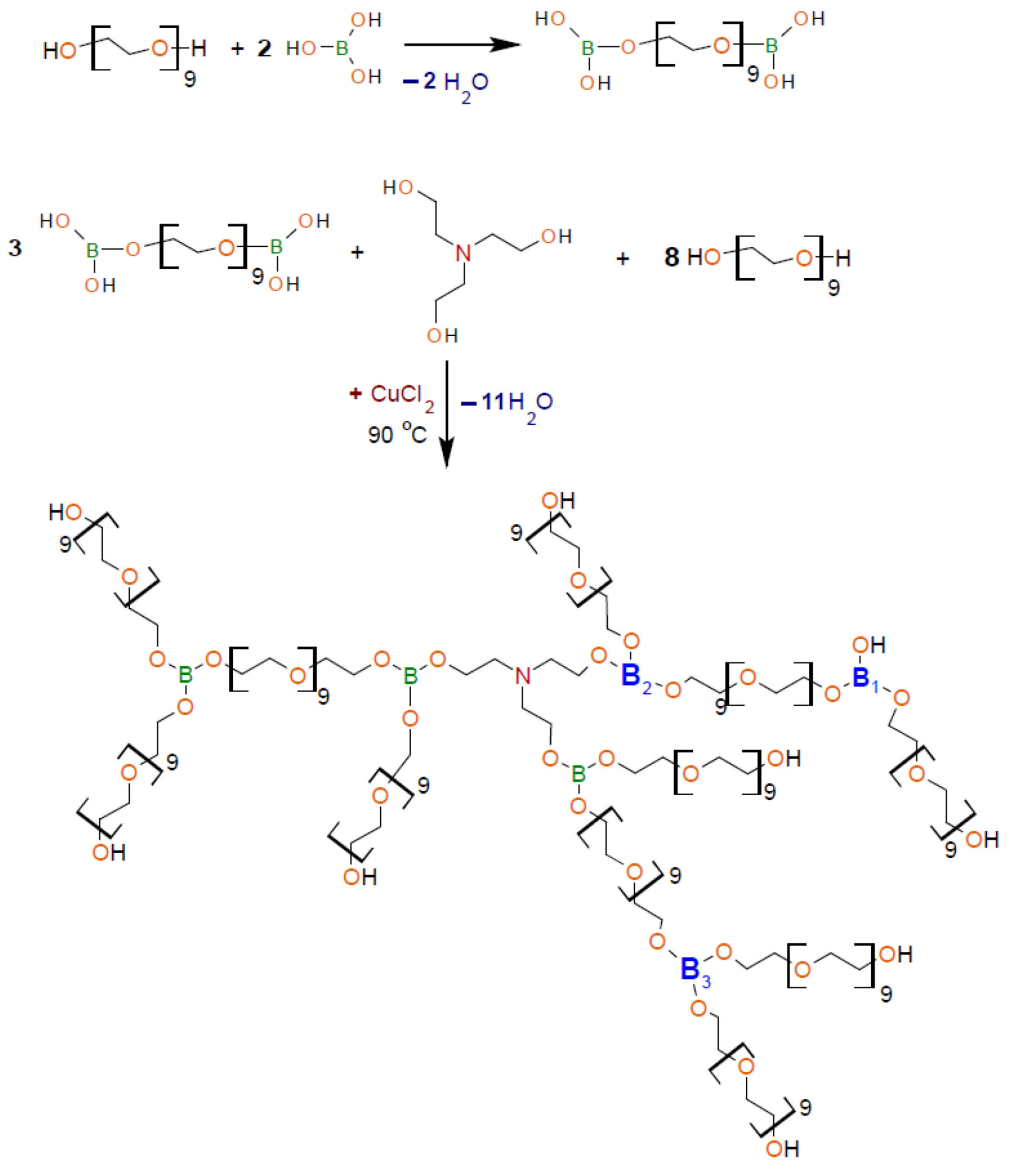
Formation of AEBA.

**Figure 2:**
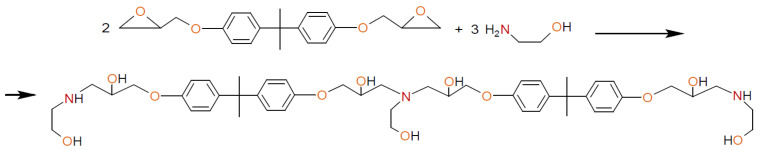
Scheme of EM synthesis.

**Figure 3:**
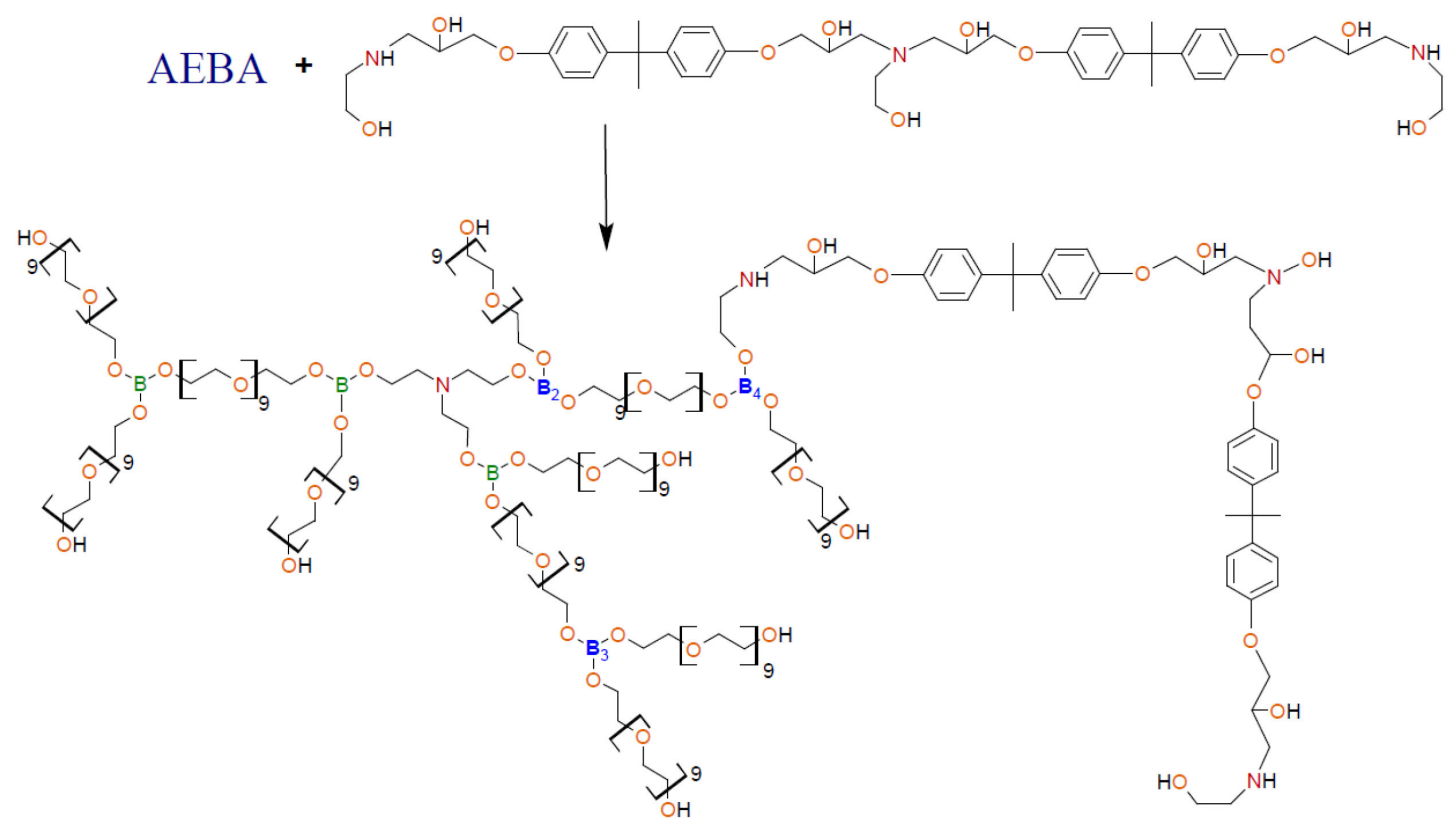
Synthesis of AEBA-EM.

To obtain a hyperbranched AEBA structure (Figure 1), the synthesis was carried out in two stages. At the first stage, a boric acid ether was synthesized. In the second stage, the resulting boric acid ether was mixed with TEA and PEG. Copper chloride was introduced into the reaction system as a catalyst. Then, EM (Figure 2) was interacted with AEBA at free B-OH groups (Figure 3).

**Figure 4:**
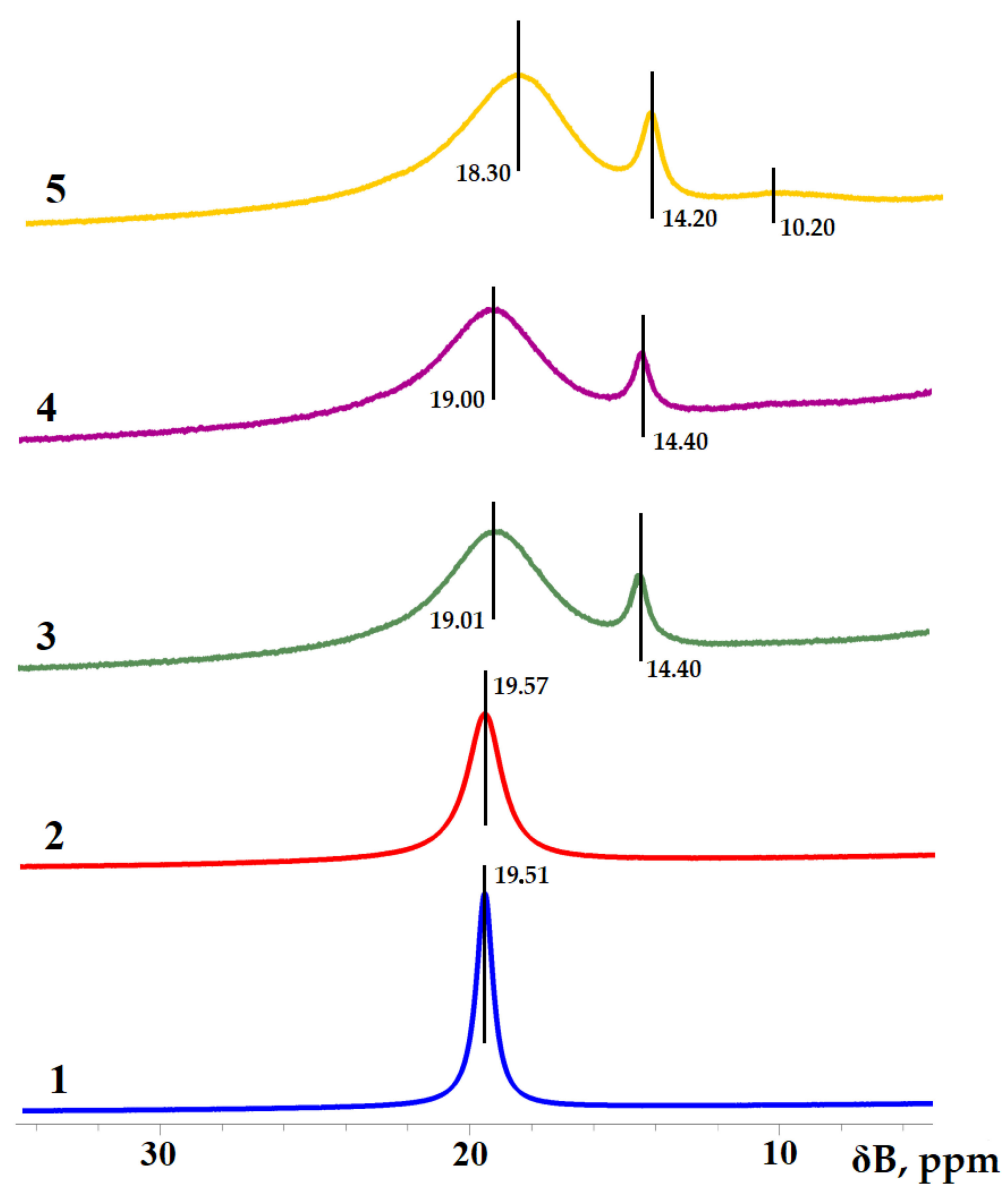
^11^B NMR spectra of compounds obtained at the following molar ratios: 1—[H_3_BO_3_]:[PEG] = 1:1 (19.51 ppm); 2—[H_3_BO_3_]:[PEG] = 1:3 (19.57 ppm); 3—[TEA]:[H_3_BO_3_]:[PEG] = 1:6:12 (AEBA) (19.01 ppm; 14.40 ppm); 4—[TEA]:[H_3_BO_3_]:[PEG] = 1:6:12 + 0.1% EM (AEBA-0.1 EM) (19.00 ppm; 14.40 ppm); 5—[TEA]:[H_3_BO_3_]:[PEG] = 1:6:12 + 2.0% EM (AEBA-2.0 EM) (18.30 ppm; 14.20 ppm; 10.20 ppm).

Aminoethers of boric acid were studied by ^11^B NMR spectroscopy (Figure 4). According to the data given in [68,69], a chemical shift of the boron atom in boric acid is 20.04 ppm. In the present study, the chemical shifts of boron atoms in the structure of the AEBA were determined from ^11^B NMR spectra of different samples: solution of H_3_BO_3_ in PEG ([H_3_BO_3_]:[PEG] = 1:1), boric acid ether obtained at [H_3_BO_3_]:[PEG] = 1:3, and aminoether of boric acid obtained at a molar ratio of [TEA]:[H_3_BO_3_]:[PEG] = 1:6:12 (AEBA). ^11^B NMR spectra of a solution of H_3_BO_3_ in PEG, ^11^B NMR spectra of boric acid ether obtained at [H_3_BO_3_]:[PEG] = 1:3 and of aminoether of boric acid obtained at a molar ratio of [TEA]:[H_3_BO_3_]:[PEG] = 1:6:12 (AEBA) were measured. The ^11^B NMR spectrum of boric acid dissolved in PEG shows a signal with a chemical shift of 19.51 ppm. For the boric acid ether obtained at [H_3_BO_3_]:[PEG] = 1:3, one broad signal is observed at 19.57 ppm, including boron atoms in partially etherified boric acid, in which the part of B–OH groups remains free (B_1_, Figure 1) and boron atoms included in fully etherified boric acid (B_3_) [48]. When triethanolamine is used for the etherification of boric acid along with PEG in the synthesis of AEBA, a boron atom (B_2_) is formed, which is located in the central site of the AEBA structure, close to the nitrogen atom. The boron atom B_2_ has a signal with the chemical shift of 14.40 ppm. In this case, the 19.51 ppm signal is shifted to 19.01 and its broadening is even greater. With an increase in the content of the EM adduct in the AEBA-EM structure, the signal at 19.57 ppm shifts even more noticeably (up to 18.30 ppm), but still remains in the low-field limit of the spectrum and is easily recognized. For AEBA-2.0 EM, there is also an increase in signal intensity at 14.20 ppm. In addition, the spectra of AEBA-2.0 EM show a weak broad signal at 10 ppm due to the boron atom (B_4_) adjacent to the EM embedded in the AEBA structure. Thus, measurements of ^11^B NMR spectra suggest that the EM adduct is embedded in the AEBA structure, forming AEBA-EM.

**Figure 5:**
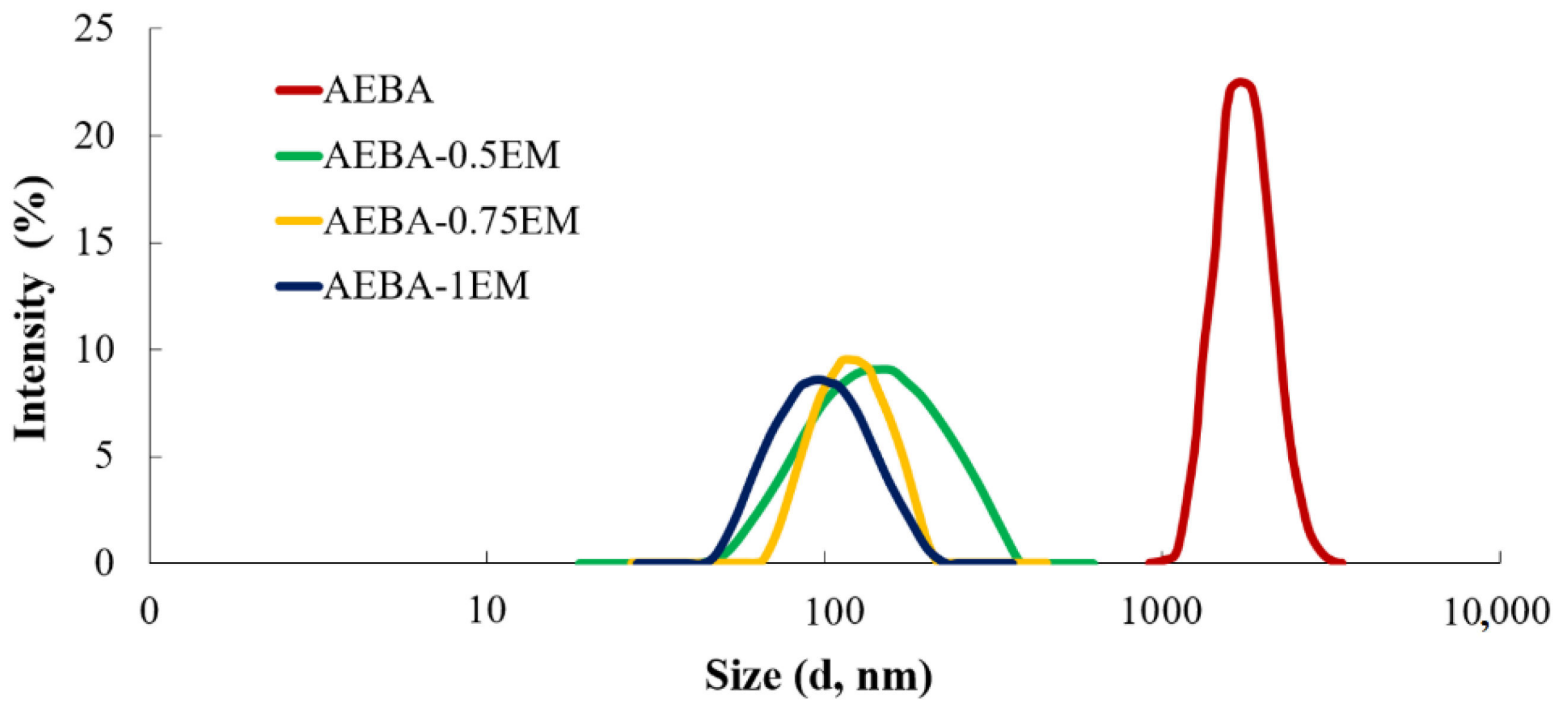
Particle size distribution of AEBA and AEBA-EM, determined by DLS.

Using the dynamic light scattering method, the influence of the EM content on the particle size of AEBA-EM was studied (Figure 5). It turned out that the use of EM leads to a significant reduction in the particle size of AEBA-EM compared to the size of unmodified AEBA. This can be explained by the fact that the association of terminal hydroxyl groups can lead to AEBA clustering. The EM adduct, being a bulk compound, partially breaks associative interactions, and the sizes of clusters into which AEBA are combined decrease.

**Figure 6:**
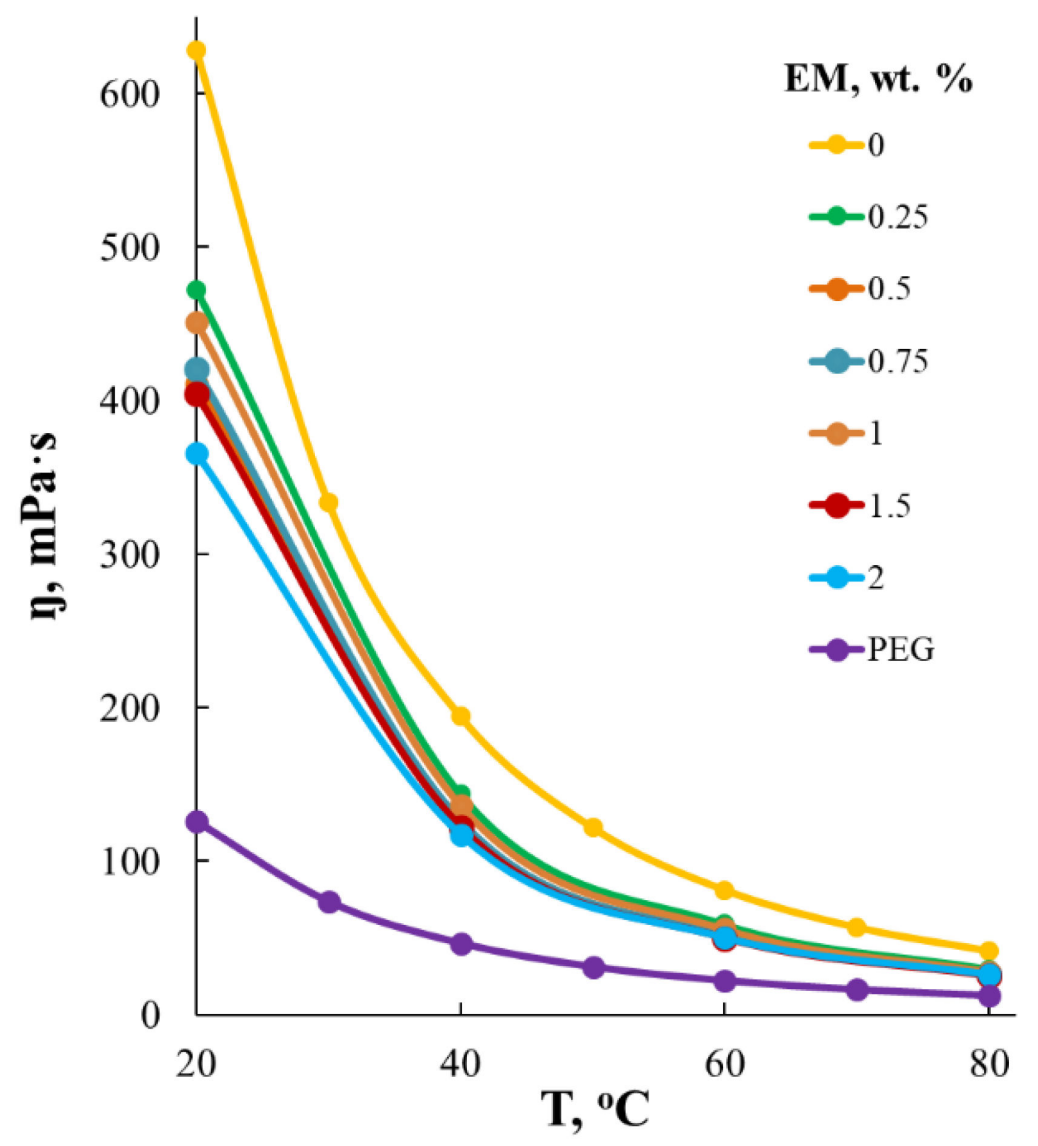
Temperature dependencies of dynamic viscosity (η, mPa·s) of AEBA, AEBA-EM and PEG.

The data obtained using the dynamic light scattering method correlate with dynamic viscosity measurements (Figure 6). The fact that the supramolecular structure of AEBA-EM differs from the method of packing of AEBA molecules is evidenced by a significant difference not only in the values of their dynamic viscosity, but also in the shape of the temperature dependence curves. According to the drop in the size of AEBA-EM particles, with an increase in the content of EM in their composition, the values of their dynamic viscosity also decrease. In addition, a feature of the AEBA and AEBA-EM clusters is their significantly higher viscosity compared to the PEG used in the synthesis of these compounds.

### 3.2. Synthesis of Polyurethanes Based on AEBA and AEBA-EM

**Figure 7:**
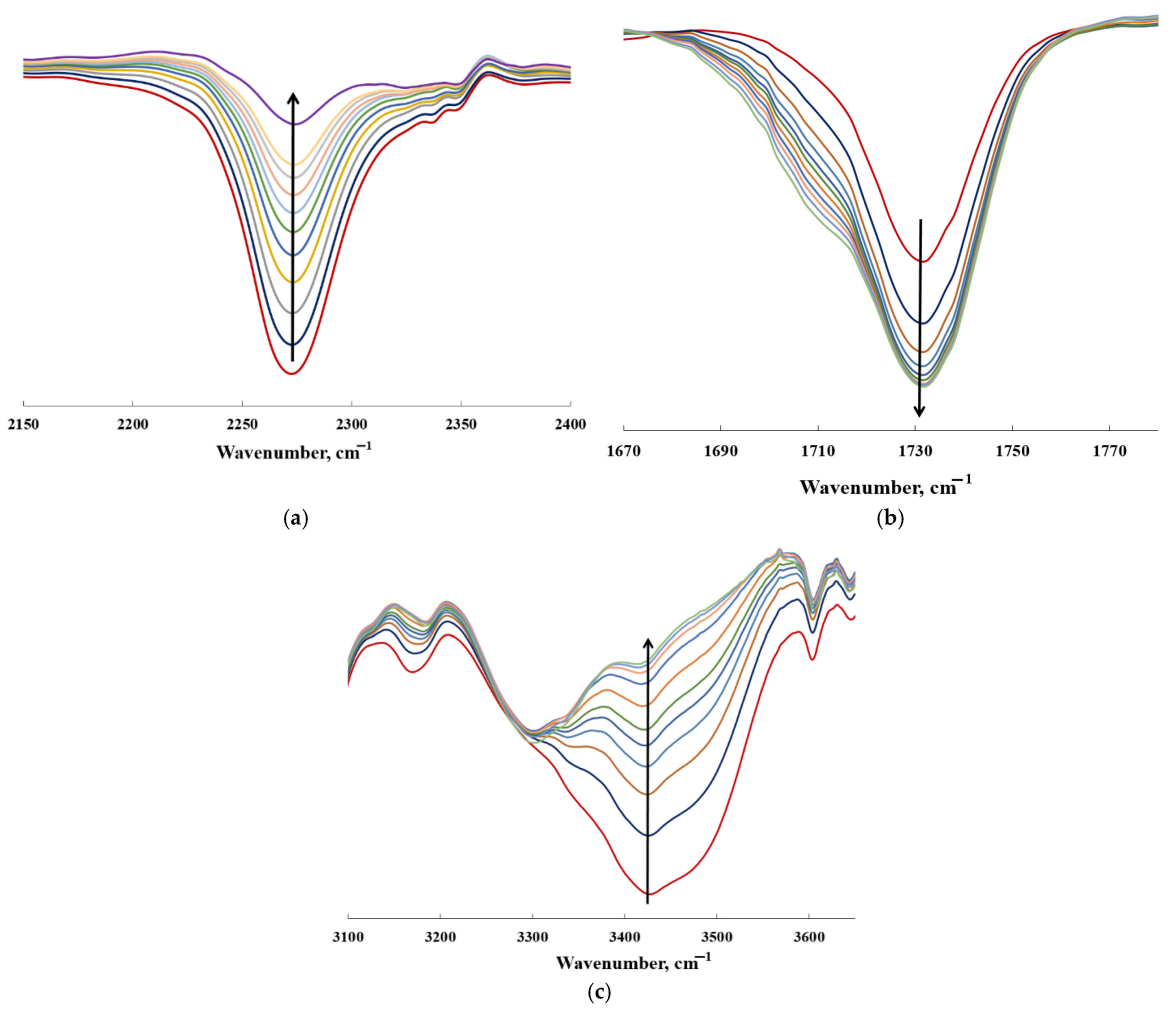
FTIR spectra measured during the interaction of AEBA with TDI. The cursor indicates changes in the spectrum consumption of isocyanate groups (**a**), formation of urethane groups (**b**) and consumption of hydroxyl groups (**c**) from the beginning of the reaction (zero minute) to its end. The time interval between measurements was 10 min. T = 25 °C.

Polyurethane film materials were synthesized based on AEBA/AEBA-EM and polyisocyanate. To study the features of the interaction of AEBA with aromatic isocyanates, 2,4-toluene diisocyanate was used, and FTIR spectroscopy was used as a research method. Due to the excessively high intensity of the analytical band at 2275 cm^−1^ used to determine the isocyanate groups, the beginning of the measurement of the FTIR spectra was 10 min from the start of the interaction. According to Figure 7a, almost complete consumption of isocyanate groups is observed. In the initial period of the reaction, an analytical band grows in the region of 1732 cm^−1^ due to stretching vibrations of the free carbonyl of the urethane group (Figure 7b). Sometime after the start of the reaction, a shoulder appears and grows in the region of 1710 cm^−1^, due to intermolecular interactions involving the carbonyl of the urethane group.

Free hydroxyl groups in the FTIR spectra appear as absorption bands in the region of 3480 cm^−1^, dimer associates at 3430 cm^−1^, and the absorption region of hydroxyl groups combined into trimers shifts to 3350 cm^−1^ (Figure 7c). During the interaction of TDI with AEBA, all hydroxyl groups are consumed.

### 3.3. Vapor Permeability and Pervaporation Characteristics of AEBA-PU and AEBA-EM-PU Membranes

**Figure 8:**
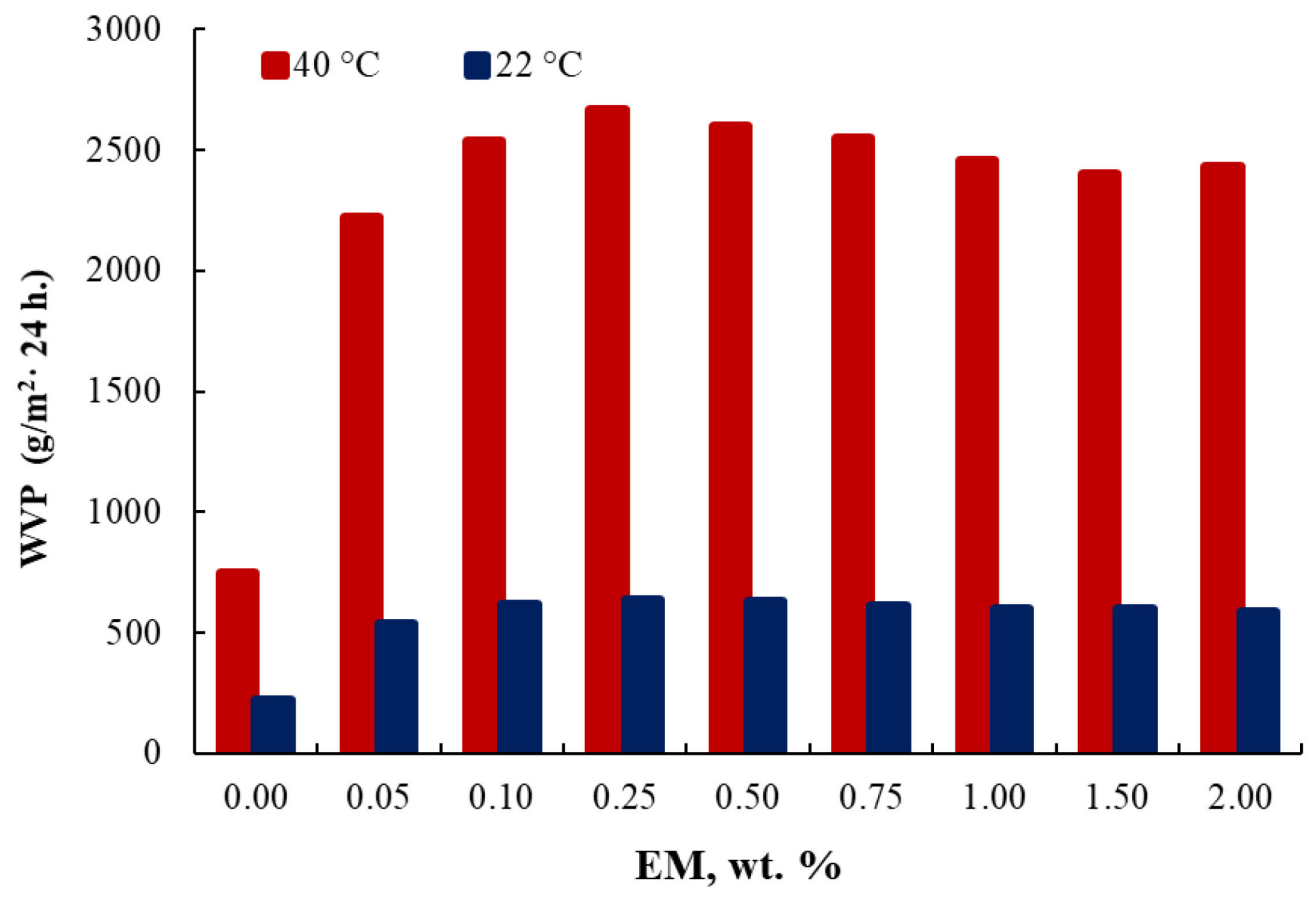
Dependences of the water vapor permeability coefficients at 22 °C and 40 °C for AEBA-PU and AEBA-EM-PU on the content of EM in AEBA-EM, wt.%.

The obtained polyurethanes were investigated as vapor-permeable and pervaporation membrane materials. According to Figure 8, the introduction of EM into the composition of AEBA leads to a threefold increase in the vapor permeability of polyurethanes synthesized based on them.

Such a significant increase in vapor permeability may be due to the peculiarity of the macromolecular packaging of AEBA and AEBA-EM. As shown by the results of studying the particle sizes of AEBA and AEBA-EM (Figure 5), AEBA molecules are combined into clusters. Modification of the structure of AEBA using EM leads to a significant reduction in the particle size of AEBA-EM relative to AEBA. A decrease in the size of clusters leads to loosening of their dense packing. Areas of clustering due to associative interactions of hydroxyl groups, together with the hydrophilic nature of polyoxyethylene glycol, create a zone through which water molecules can penetrate. However, after EM content exceeding 0.5 wt.%, the values of the vapor permeability coefficient drop slightly.

The values of water contact angles and water absorption of AEBA-EM-PU at different EM content (Table 1) are consistent with the patterns of change in vapor permeability coefficients. Thus, an increase in vapor permeability is accompanied by a decrease in the values of the contact angles and an increase in the water absorption of AEBA-EM-PU. A decrease in vapor permeability, an increase in the water contact angles and an increase in water absorption of AEBA-EM-PU after an EM content of more than 0.5 wt.% is a consequence of the fact that under such conditions, along with the destruction of associative interactions, the structuring effect of EM on AEBA-EM and, accordingly, on AEBA-EM-PU is showing up more and more.

That is, at a low content of EM in the composition of AEBA-EM, its main effect on the properties of AEBA-EM-PU is its effect on clustering processes, as a result of which the terminal hydroxyl groups of AEBA enter into associative interactions that prevent the formation of free space in the polymer matrix. The use of the EM adducts for AEBA structuring leads to a partial destruction of associative interactions. As a result, the free space in the hydrophilic environment increases in the polyurethane matrix. An increase in the content of the EM has an additive effect on the studied properties of polyurethanes. However, as the content of the adduct increases, the dependencies cease to be additive due to the fact that, along with the destruction of associative interactions, the EM begins to exert an additional influence on the formation of the polymer network.

The obtained polyurethanes were investigated as pervaporation membranes for the isopropanol dehydration.

To describe the process of mass transfer through a polymer membrane, the solution-diffusion model is usually used. According to this model, the components of the mixture being separated are adsorbed or dissolved on one membrane surface, diffused in the membrane, and the components are removed from the membrane surface into the permeate volume. The mass flow of component i for the pervaporation process will be as follows:

(5) $$j_{i}=K_{i}\frac{\left(P_{i}^{F}-P_{i}^{p}\right)}{\delta_{m}}$$

where $P_{i}^{F},P_{i}^{P}$ are partial pressure of the penetrating component i at the membrane interface in solution and in the permeate, respectively; $\delta_{m}$ is the membrane thickness; K_i_ is the permeability coefficient which takes into account both the amount of adsorption of component i on the membrane and the rate of diffusion inside the membrane. The permeability coefficient is an important value that characterizes the kinetic and separation characteristics of the membrane material, the value of which determines the required surface area.

The experimental data obtained in this work make it possible to calculate K for water:

(6) $$K_{W}=\frac{J\delta_{m}x_{W}^{P}}{\left(P_{W}^{S}\gamma_{W}^{}x_{W}^{F}-P^{P}x_{W}^{P}\right)}$$

where $P_{W}^{S}$ is the vapor pressure of pure water at process temperature; $X_{W}^{F}$ is the mole fraction of water in the retant, we assume that there is no concentration polarization; $\gamma_{W}$ is the water activity coefficient in the liquid phase, calculated according to the Wilson equation [70]; $P_{W}^{S}$ is the mole fraction of water in permeate; and $P^{P}$ is the pressure in the permeate region, which in all experiments was 20 mm Hg.

The calculated values of the penetration coefficient are presented in Table 2. It can be seen that in all cases with increasing temperature it either slightly decreases or does not change. Given that the diffusion coefficient, as a rule, increases exponentially with increasing temperature, the decrease in the permeation coefficient should be associated with a significant drop in the amount of water sorption on the membrane.

The pervaporation coefficient and flux of pervaporation membranes based on AEBA-EM-PU are significantly higher compared to membranes based on AEBA-PU. It should also be noted that the increase in permeation and flux values correlates with the patterns of increase in vapor permeability measured for AEBA-EM-PU.

The total time of experimental studies carried out on one membrane averaged 30 h. At the same time, returning to the initial conditions of the experiment, we observed the reproducibility of the measured fluxes and selectivity.

Results obtained on pervaporation characteristics, in comparison with other polyurethane systems, are characterized by a significantly higher flux value and have comparable selectivity indicators at a similar concentration of separated mixtures [30,31,32,33].

**Figure 9:**
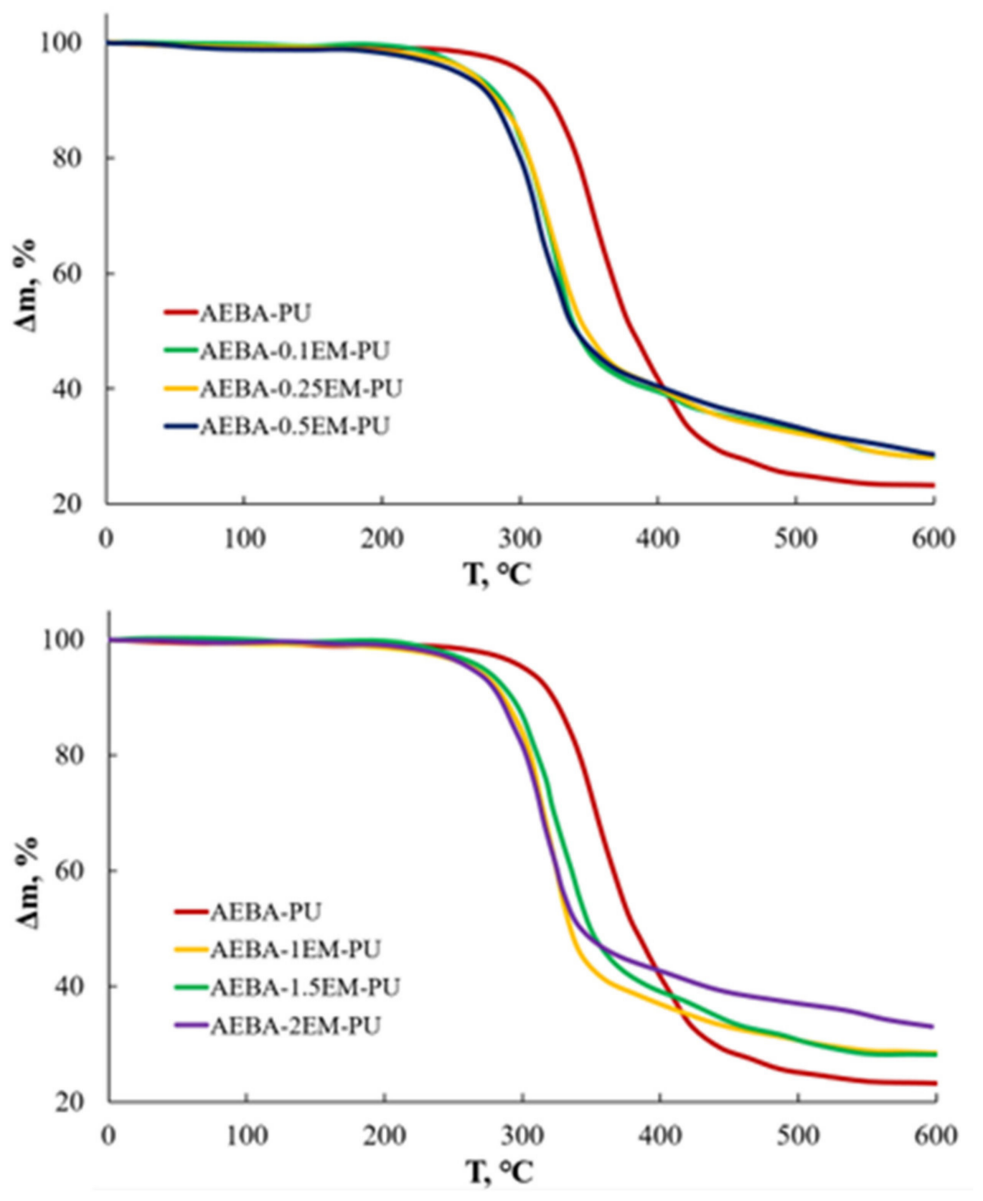
TGA curves for AEBA-PU and AEBA-EM-PU under nitrogen.

A decrease in the packing density of cluster structures and the appearance of hydrophilic channels through which the movement of water molecules is intensified caused a decrease in the thermal stability of AEBA-EM-PU relative to AEBA-PU (Figure 9). However, increasing the content of EM in the AEBA-EM composition leads to a noticeable increase in the content of carbon residue during thermal decomposition of AEBA-EM-PU.

**Figure 10:**
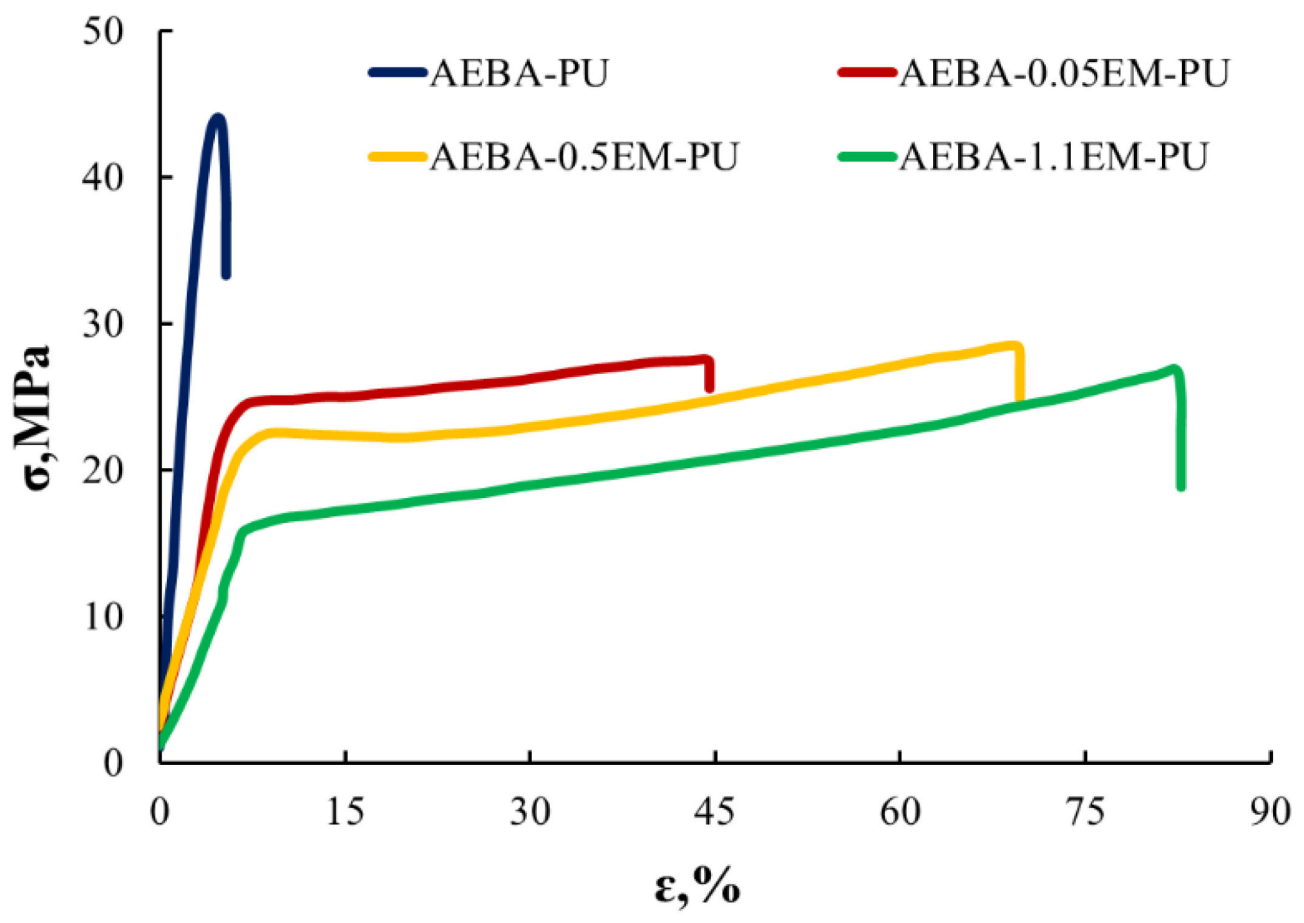
Tensile tests of AEBA-PU and AEBA-EM-PU.

A decrease in the size of clusters entails a change in the physical and mechanical behavior of AEBA-EM-PU (Figure 10). It can be concluded that a decrease in the size of clusters and the appearance of hydrophilic channels leads to loosening of the supramolecular structure of AEBA-EM-PU and an increase in the free volume of polyurethanes. All these factors cause AEBA-EM-PU to decrease in strength relative to AEBA-PU. An increase in elongation at break is a consequence of an increase in the number of labile intermolecular interactions due to the existence of numerous associative interactions in the composition of AEBA-EM-PU.

## 4. Conclusions

On the basis of aminoethers of boric acid, polyurethane vapor-permeable and pervaporative membranes were obtained. AEBAs, the structure of which is modified by bulk adducts of diphenylol propane diglycidyl ether and ethanolamine, were studied. It turned out that AEBA exists in the form of clusters, and the use of EM as a result of partial destruction of associative interactions leads to a significant decrease in the size of AEBA-EM particles and their viscosity compared to unmodified AEBA.

The introduction of EM into the composition of AEBA leads to a threefold increase in the vapor permeability of polyurethanes obtained on their basis. The observed effect is explained by the fact that a decrease in the size of clusters leads to loosening of their dense packing. Areas of clustering due to associative interactions of hydroxyl groups, together with the hydrophilic nature of polyoxyethylene glycol, create channels through which water molecules can penetrate. It was found that the increase in the permeability coefficients during pervaporative dehydration of isopropanol correlates with the patterns of the increase in water vapor permeability coefficients.

## Figures and Tables

**Table 1:** Water contact angles and water absorption of AEBA-EM-PU at various EM content.

EM Content, wt.%	Water Contact Angle	Water Absorption, wt.%
0.0	85	12.0
0.05	78	13.5
0.1	66	14.3
0.25	45	15.0
0.5	43	15.5
1.0	70	16.5
1.5	72	17.2
2.0	72	17.5

**Table 2:** Isopropanol dehydration using pervaporation membranes based on AEBA-PU and AEBA-EM-PU.

Polyurethane	Concentration IPA, wt.%	IPA in Permeate, wt.%	* Flux, g/m²∙h	Separation Factor	Activity, $\gamma_{W}$	K_W_, g/(m∙s∙Pa)
Feed temperature, 40 °C						
AEBA-PU	95	6.0	21	295	4.09	0.29
	85	2.9	44	190	3.21	0.19
AEBA-0.25EM-PU	95	17.4	128	90	4.09	1.47
	85	13.6	352	36	3.21	1.36
Feed temperature, 60 °C						
AEBA-PU	95	21.7	77	69	3.62	0.20
	85	11.4	148	44	2.94	0.19
AEBA-0.25EM-PU	95	30.7	449	43	3.62	1.03
	85	16.2	1067	29	2.94	1.30

* Flow rate is based on membrane thickness δ_m_ = 10 µm.

## Data Availability

The data presented in this study are available on request from the corresponding author.

## References

[1] Scott K. (1995). Separation of liquid mixtures/pervaporation. Handbook of Industrial Membranes.

[2] Burts K.S., Plisko T.V., Bildyukevich A.V., Li G., Kujawa J., Kujawski W. (2022). Development of dynamic PVA/PAN membranes for pervaporation: Correlation between kinetics of gel layer formation, preparation conditions, and separation performance. Chem. Eng. Res. Des..

[3] Xi T., Lu Y., Ai X., Tang L., Yao L., Hao W., Cui P. (2019). Ionic liquid copolymerized polyurethane membranes for pervaporation separation of benzene/cyclohexane mixtures. Polymer.

[4] Amaral R.A., Habert R.A., Borges C.P. (2014). Activated carbon polyurethane membrane for a model fuel desulfurization by pervaporation. Mater. Lett..

[5] Das S., Banthia A.K., Adhikari B. (2008). Porous polyurethane urea membranes for pervaporation separation of phenol and chlorophenols from water. Chem. Eng. J..

[6] Tonga Z., Liu X., Zhang B. (2020). Sulfonated graphene oxide based membranes with enhanced water transport capacity for isopropanol pervaporation dehydration. J. Membr. Sci..

[7] Tseng C., Liu Y.-L. (2022). Creation of water-permeation pathways with matrix-polymer functionalized carbon nanotubes in polymeric membranes for pervaporation desalination. J. Membr. Sci. Lett..

[8] Zhai Y., Zhang B., Liu X., Tong Z. (2020). Manipulation of homogeneous membranes with nano-sized spherical polyelectrolyte complexes for enhanced pervaporation performances in isopropanol dehydration. Sep. Purif. Technol..

[9] Liu S., Zhou G., Cheng G., Wang X., Liu G., Jin W. (2022). Emerging membranes for separation of organic solvent mixtures by pervaporation or vapor permeation. Sep. Purif. Technol..

[10] Kamtsikakis A., Delepierre G., Weder C. (2021). Cellulose nanocrystals as a tunable nanomaterial for pervaporation membranes with asymmetric transport properties. J. Membr. Sci..

[11] Liu G., Jin W. (2021). Pervaporation membrane materials: Recent trends and perspectives. J. Membr. Sci..

[12] Tang S., Dong Z., Zhu X., Zhao Q. (2019). A poly(ionic liquid) complex membrane for pervaporation dehydration of acidic water-isopropanol mixtures. J. Membr. Sci..

[13] Jiang L.Y., Wang Y., Chung T.-S., Qiao X.Y., Lai J.-Y. (2009). Polyimides membranes for pervaporation and biofuels separation. Prog. Polym. Sci..

[14] Ong Y.K., Wang H., Chung T.-S. (2012). A prospective study on the application of thermally rearranged acetate-containing polyimide membranes in dehydration of biofuels via pervaporation. Chem. Eng. Sci..

[15] Dmitrenko M., Penkova A., Kuzminova A., Missyul A., Ermakov S., Roizard D. (2018). Development and Characterization of New Pervaporation PVA Membranes for the Dehydration Using Bulk and Surface Modifications. Polymers.

[16] Toth A.J., Andre A., Haaz E., Mizsey P. (2015). New horizon for the membrane separation: Combination of organophilic and hydrophilic pervaporations. Sep. Purif. Technol..

[17] Feng X., Huang R.Y.M. (1997). Liquid Separation by Membrane Pervaporation: A Review. Ind. Eng. Chem. Res..

[18] Hung W.-S., Chang S.-M., Lecaros R.L.G., Ji Y.-L., An Q.-F., Hu C.-C., Lee K.-R., Lai J.-Y. (2017). Fabrication of hydrothermally reduced graphene oxide/chitosan composite membranes with a lamellar structure on methanol dehydration. Carbon.

[19] Zhang H., Wang Y. (2016). Poly(vinyl alcohol)/ZIF-8-NH2mixed matrix membranes for ethanol dehydration via pervaporation. AIChE J..

[20] Xia L.L., Li C.L., Wang Y. (2016). In-situ crosslinked PVA/organosilica hybrid membranes for pervaporation separations. J. Membr. Sci..

[21] Le N.L., Wang Y., Chung T.S. (2011). Pebax/POSS mixed matrix membranes for ethanol recovery from aqueous solutions via pervaporation. J. Membr. Sci..

[22] Le N.L., Tang Y.P., Chung T.-S. (2013). The development of high-performance 6FDA-NDA/DABA/POSS/Ultem dual-layer hollow fibers for ethanol dehydration via pervaporation. J. Membr. Sci..

[23] Król P., Król B. (2020). Structures, properties and applications of the polyurethane ionomers. J. Mater. Sci..

[24] Chen S.H., Yu K.C., Houng S.L., Lai J.Y. (2000). Gas transport properties of HTPB based polyurethane/cosalen membrane. J. Membr. Sci..

[25] Gupta T., Pradhan N.C., Adhikari B. (2002). Synthesis and performance of a novel polyurethaneurea as pervaporation membrane for the selective removal of phenol from industrial waste water. Bull. Mater. Sci..

[26] Tsai M.H., Huang S., Liu S., Chen C., Chen P., Chen S. (2008). Synthesis and properties of poly(urethane-imide) interpenetrating network membranes. Desalination.

[27] Nunez C.M., Chiou B.S., Andrady A., Khan S.A. (2000). Solution rheology of hyperbranched polyesters and their blends with linear polymers. Macromolecules.

[28] Liou Y.J., Chin T.M., Kuo Y.M., Chao D.Y. (2006). Water vapor-permeable polyurethane ionomer. J. Appl. Polym. Sci..

[29] Astruc D., Boisselier E., Ornelas C. (2010). Dendrimers Designed for Functions: From Physical, Photophysical, and Supramolecular Properties to Applications in Sensing, Catalysis, Molecular Electronics, Photonics, and Nanomedicine. Chem. Rev..

[30] Huang S., Chang P., Tsai M., Chang H. (2007). Properties and pervaporation performances of crosslinked HTPB-based polyurethane membranes. Sep. Purif. Technol..

[31] Chao M.-S., Huang S.-L. (2005). Epoxidized HTPB-based polyurethane membranes for pervaporation separation. J. Chin. Chem. Soc..

[32] Tsai M.-H., Huang S.-L., Chang P.-H., Chen C.-J. (2007). Properties and pervaporation separation of hydroxyl-terminated polybutadiene-based polyurethane/poly(methyl metharcylate) interpenetrating networks membranes. J. Appl. Polym. Sci..

[33] Das S., Sarkar S., Basak P., Adhikari B. (2008). Dehydration of alcohols by pervaporation using hydrophilic polyether urethane membranes. J. Sci. Ind. Res. India..

[34] Tsai L., Chen Y. (2008). Hyperbranched Poly(fluorenevinylene)s Obtained from Self-Polymerization of 2,4,7-Tris(bromomethyl)-9,9-dihexylfluorene. Macromolecules.

[35] Kricheldorf H.R., Hobzova R., Vakhtangishvili L.V., Schwarz G. (2005). Multicyclic Poly(ether ketone)s Obtained by Polycondensation of 2,6,4-Trifluorobenzophenone with Various Diphenols. Macromolecules.

[36] Satija J., Karunakaran B., Mukherji S. (2014). A dendrimer matrix for performance enhancement of evanescent wave absorption-based fiber-optic biosensors. RSC Adv..

[37] Irfan M., Seiler M. (2010). Encapsulation using hyperbranched polymers: From research and technologies to emerging applications. Ind. Eng. Chem. Res..

[38] Deng Y., Saucier-Sawyer J.K., Hoimes C.J., Zhang J., Seo Y.-E., Andrejecsk J.W., Saltzman W.M. (2014). The effect of hyperbranched polyglycerol coatings on drug delivery using degradable polymer nanoparticles. Biomaterials.

[39] Fritz T., Hirsch M., Richter F.C., Müller S.S., Hofmann A.M., Rusitzka K.A., Markl J., Massing U., Frey H., Helm M. (2014). Click modification of multifunctional liposomes bearing hyperbranched polyether chains. Biomacromolecules.

[40] Davletbaeva I.M., Sazonov O.O., Fazlyev A.R., Davletbaev R.S., Efimov S.V., Klochkov V.V. (2019). Polyurethane ionomers based on amino ethers of orto-phosphoric acid. RSC Adv..

[41] Davletbaeva I.M., Sazonov O.O., Nikitina E.A., Kapralova V.M., Nizamov A.A., Akhmetov I.G., Arkhipov A.V., Sudar N.T. (2022). Dielectric Properties of Organophosphorus Polyurethane Ionomers. J. Appl. Polym. Sci..

[42] Jiang G., Chen W., Xia W. (2008). Environmental-Sensitive Hyperbranched Polymers as Drug Carriers. Des. Monomers Polym..

[43] Zhang K., Li L., Yu W., Hu M., Zhou Y., Fan X. (2017). Synthesis of HTPB based PU-PS IPN membrane for pervaporation recovery of butanol. J. Wuhan Univ. Technol. Mater. Sci. Edit..

[44] Huang J., Meagher M.M. (2001). Pervaporative recovery of n-butanol from aqueous solutions and ABE fermentation broth using thin-film silicalite-filled silicone composite membranes. J. Membr. Sci..

[45] Chapman P.D., Oliveira T., Livingston A.G., Li K. (2008). Membranes for the dehydration of solvents by pervaporation. J. Membr. Sci..

[46] Hoshi M., Kogure M., Saitoh T., Nakagawa T. (1997). Separation of aqueous phenol through polyurethane membranes by pervaporation. J. Appl. Polym. Sci..

[47] Davletbaeva I.M., Sazonov O.O., Zakirov I.N., Gumerov A.M., Klinov A.V., Fazlyev A.R., Malygin A.V. (2021). Organophosphorus polyurethane ionomers as water vapor permeable and pervaporation membranes. Polymers.

[48] Davletbaeva I.M., Nurgaliyeva G.R., Akhmetshina A.I., Davletbaev R.S., Atlaskin A.A., Sazanova T.S., Efimov S.V., Klochkov V.V., Vorotyntsev I.V. (2016). Porous polyurethanes based on hyperbranched amino ethers of boric acid. RSC Adv..

[49] Davletbaeva I.M., Dulmaev S.E., Sazonov O.O., Gumerov A.M., Davletbaev R.S., Valiullin L.R., Ibragimov R.G. (2020). Polyurethane based on modified aminoethers of boric acid. Polym. Sci. Ser. B.

[50] Davletbaeva I.M., Emelina O.Y., Vorotyntsev I.V., Davletbaev R.S., Grebennikova E.S., Petukhov A.N., Ahkmetshina A.I., Sazanova T.S., Loskutov V.V. (2015). Synthesis and properties of novel polyurethanes based on amino ethers of boric acid for gas separation membranes. RSC Adv..

[51] Aydoğmuş N., Köse D.A., Beckett M.A., Karan B.Z. (2014). Organic biomolecules bind to phosphate through borate linkages in aqueous solutions. Turk. J. Chem..

[52] Tevyashova A.N., Chudinov M.V. (2021). Progress in the medicinal chemistry of organoboron compounds. Russ. Chem. Rev..

[53] Bondarenko S.N., Khohlova T.V., Orlova S.A., Tuzhikov O. (2006). Synthesis of oligomeric boron-containing phosphopolyols. Russ. J. Gen. Chem..

[54] Shteinberg L.Y. (2011). A study of the kinetics and mechanism of amidation of 4-nitrobenzoic acid with ammonia, catalyzed by boric acid in the presence of polyethylene glycol PEG-400. Russ. J. Appl. Chem..

[55] Androshchuk A., Lenskii M.A., Belousov A.M. (2011). The Interaction of Polyesters and Polymethylene Esters of Phenols and Boric Acid with Epoxy Resin. Int. Polym. Sci. Technol..

[56] Zhang Z., Mao J., Zhang Y., Han T., Yang B., Xiao W. (2019). Improved fracturing fluid using: Organic amino boron composite crosslinker with B-N covalent bond. J. Appl. Polym. Sci..

[57] Tanaka K., Chujo Y. (2012). Advanced luminescent materials based on organoboron polymers. Macromol. Rapid Commun..

[58] Vedelago J., Mattea F., Triviño S., Montesinos M.D., Keil W., Valente M., Romero M.R. (2021). Smart material based on boron crosslinked polymers with potential applications in cancer radiation therapy. Sci. Rep..

[59] Dai K., Zheng Y., Wei W. (2021). Organoboron-Containing Polymer Electrolytes for High-Performance Lithium Batteries. Adv. Funct. Mater..

[60] Paciorek-Sadowska J., Czuprynski B., Liszkowska J. (2015). Boron-containing fire retardant rigid polyurethane-polyisocyanurate foams: Part I—Polyol precursors based on boric acid and di(hydroxymethyl)urea derivatives. J. Fire Sci..

[61] Shirke A.G., Dholakiya B.Z., Kuperkar K. (2017). Modification of tung oil-based polyurethane foam by anhydrides and inorganic content through esterification process. J. Appl. Polym. Sci..

[62] Liu X., Hong W., Chen X. (2020). Continuous Production of Water-Borne Polyurethanes: A Review. Polymers.

[63] Zarzyka I. (2016). Preparation and characterization of rigid polyurethane foams with carbamide and borate groups. Polym. Int..

[64] Czupryński B., Paciorek J.W. (1999). The effect of tri(2-hydroxypropyl) borate on the properties of rigid polyurethane-polyisocyanurate foams. Polimery.

[65] Łukasiewicz B., Lubczak J. (2014). Polyurethane foams with purine ring and boron. J. Cell. Plast..

[66] Chmiel E., Lubczak J. (2019). Polyurethane foams with 1,3,5-triazine ring, boron and silicon. J. Cell. Plast..

[67] Qiu F., Zhao W., Han S., Zhuang X., Lin H., Zhang F. (2016). Recent Advances in Boron-Containing Conjugated Porous Polymers. Polymers.

[68] Chen X., Chew S.L., Kerton F.M., Yan N. (2014). Direct conversion of chitin into a N-containing furan derivative. Green Chem..

[69] Cai L., Lim H., Fitzkee N.C., Cosovic B., Jeremic D. (2020). Feasibility of Manufacturing Strand-Based Wood Composite Treated with β-Cyclodextrin–Boric Acid for Fungal Decay Resistance. Polymers.

[70] Reid R.C., Prausnitz J.M., Sherwood T.K. (1977). The Properties of Gases and Liquids.

